# Research on Formulation Optimization and Storage Stability of *Pueraria lobata* Compound Beverage: Flavor Analysis and Shelf-Life Prediction

**DOI:** 10.3390/molecules31111798

**Published:** 2026-05-23

**Authors:** Zaixiang Lou, Xinyan Cui, Beiqi Wu, Hongxin Wang, Nattaya Konsue, Sook Wah Chan, Bing Kang

**Affiliations:** 1 State Key Laboratory of Food Science and Resources, Jiangnan University, Wuxi 214122, China; 18762628639@163.com (X.C.); bqwu@aliyun.com (B.W.); hxwang@jiangnan.edu.cn (H.W.); 2 School of Food Science and Technology, Jiangnan University, Wuxi 214122, China; 3 Food Science and Technology Program, School of Agro-Industry, Mae Fah Luang University, Chiang Rai 57100, Thailand; nattaya.kon@mfu.ac.th; 4 School of Biosciences, Faculty of Health and Medical Sciences, Taylor’s University, Subang Jaya 47500, Selangor, Malaysia; sookwah.chan@taylors.edu.my; 5 Centre for Active Living, Taylor’s University, Subang Jaya 47500, Selangor, Malaysia; 6 Jilin Academy of Chinese Medicine Sciences, Changchun 130021, China; 18604313377@163.com

**Keywords:** *Pueraria lobata*, herbal beverage, storage stability, shelf-life prediction, Zeta potential, Arrhenius kinetics, GC-MS

## Abstract

Developing a *Pueraria lobata* compound beverage is of great significance for enhancing the utilization value of *Pueraria lobata* resources. However, its flavor balance, physical stability, and quality changes during storage require further investigation. This study aimed to develop a high-quality *Pueraria lobata* compound beverage and establish a reliable shelf-life prediction model. The optimal formulation was determined using orthogonal design and multi-index evaluation, including extract stock solution, mogroside, citric acid, and a composite stabilizer consisting of xanthan gum (XG) and sodium carboxymethyl cellulose (CMC-Na). GC-MS analysis identified multiple volatile compounds collectively forming the characteristic flavor profile. During storage, physicochemical properties, sensory quality, and active component contents changed to varying extents, with deterioration significantly accelerated at higher temperatures. Among the quality indicators, Zeta potential was selected as the most suitable predictor because it showed a strong correlation with sensory scores and fitted the first-order kinetic model well. Based on the established Arrhenius-based prediction model, the predicted shelf-lives of the *Pueraria lobata* compound beverage at 4 °C, 27 °C, and 37 °C were 193, 104, and 82 days, respectively. These findings provide a solid theoretical basis for formulation design, stability improvement, and shelf-life evaluation of functional *Pueraria lobata* beverages.

## 1. Introduction

*Pueraria lobata* is a traditional medicinal and edible plant resource in China. It is rich in bioactive substances, including puerarin, daidzein, total flavonoids, and polyphenols. These compounds exhibit significant antioxidant, metabolic-regulating, and cardioprotective activities [1]. In recent years, some progress has been made in the development of *P. lobata* beverage. However, most studies have focused on extraction processes [2]. There remains a lack of systematic and in-depth investigation into the overall physicochemical stability, sensory quality, and changes in active components during storage of *P. lobata* beverages as a complex colloidal system. As consumers increasingly prefer natural and healthy diets, developing functional plant-based foods with excellent sensory quality has become a key focus in beverage research. However, high levels of polyphenols and tannins in plant extracts often impart a distinct bitter and astringent taste. Studies have shown that polyphenols interact and form complexes with salivary proteins. This reaction reduces oral lubrication and creates a dry, rough, and astringent sensation. Such sensory defects limit consumer acceptance [3]. Furthermore, plant-based beverages are complex colloidal systems. They consist of proteins, polysaccharides, and fine fiber particles. These systems are highly susceptible to instability phenomena, such as flocculation, stratification, or sedimentation, during processing and storage. This instability severely affects the physical stability and active component content of the products [4].

Mogroside, a natural and low-calorie sweetener with high relative sweetness, requires minimal concentrations to achieve desired sensory profiles, making it an ideal alternative to artificial sweeteners in food [5]. Currently, this sweetener has been launched in countries such as the United States, Canada, Japan, and China [6]. To address the aforementioned physical instability, xanthan gum (XG) and sodium carboxymethyl cellulose (CMC-Na) were selected. Hydrophilic colloids can be used as stabilizers to solve the problems of easy layering and sedimentation in plant-based beverages. XG is an anionic exopolysaccharide produced by microbial fermentation. It possesses excellent water solubility, thickening properties, and thermal stability [7]. XG forms a three-dimensional network that provides steric hindrance and increases viscosity, whereas CMC-Na enhances electrostatic repulsion and improves particle dispersion, thereby synergistically increasing overall system stability [8]. Previous studies have shown XG, CMC-Na, and their composite systems have been widely used in fruit and vegetable juice beverage, protein beverage, and herbal extracts to improve suspendability, rheological properties, and storage stability [7,9]. Beverage quality changed significantly during storage, and these changes were influenced by storage temperature and storage time. Prolonged storage can alter pH and negatively impact soluble solid content (SSC), color, stability, active component content, and sensory score. Currently, zero-order kinetics, first-order kinetics, and the Arrhenius equation are commonly used approaches for predicting food shelf-life [10]. Among them, kinetic models are commonly used to describe the change patterns of quality indicators over time, while the Arrhenius model can further reveal the effect of temperature on the reaction rate constant. Therefore, these models have been widely applied in accelerated storage studies of beverage, fruit juice, and functional food [11]. Although the above methods have been applied in various food systems, systematic studies on the quality changes and shelf-life prediction of *P*. *lobata* herbal beverage during storage remain relatively limited. In particular, there is a lack of comprehensive evaluation that integrates sensory quality, physicochemical stability, and changes in functional active components.

Traditional Chinese medicine formulas emphasize syndrome differentiation and treatment as well as the synergistic efficacy of herbal combinations [12]. Following the “monarch, minister, assistant, and guide” principle, this study constructed a medicine-food homology formula centered on *P*. *lobata*, *Hovenia dulcis*, *Ganoderma lucidum*, and *Lycium barbarum*. From the perspective of traditional theory, *P*. *lobata* and *H*. *dulcis* both possess anti-alcohol and hepatoprotective effects, serving together as the “monarch” herbs to synergistically alleviate toxicity. *G*. *lucidum* tonifies Qi and calms the mind, acting as the “minister” herb. *L*. *barbarum* nourishes the liver and kidneys, acting as the “assistant” herb. These two herbs assist in body repair and collectively enhance overall regulatory capacity. From a modern pharmacological perspective, this combination has clear synergistic targets. Studies have shown that the “*P. lobata-H. dulcis*” herb pair ameliorates alcoholic liver injury by reducing oxidative stress and inflammatory responses [13,14]. Ganoderic acid and *L*. *barbarum* polysaccharides further enhance hepatoprotective effects by regulating lipid metabolism and the gut microbiota [15,16,17].

Based on the above scientific rationale for the herbal combination, this study aims to develop a botanical beverage that possesses both traditional efficacy and modern nutritional value. Although the respective anti-alcohol and hepatoprotective effects of *P. lobata*, *H. dulcis*, *G. lucidum*, and *L. barbarum* have been extensively reported, systematic research on product development, flavor characteristics, and storage stability following their scientific combination has not yet been documented. This study is the first to apply the medicine-food homology formula of “*P. lobata-H. dulcis-G. lucidum-L. barbarum*”, grounded in both the “monarch, minister, assistant, and guide” theory and modern pharmacology, to the complete development process of a botanical beverage. Based on formula optimization, the volatile flavor components of this compound beverage were systematically characterized, and its quality changes during storage were evaluated for the first time. Through this research, we aim to promote the deep processing and high-value utilization of *P*. *lobata* resources, while providing a theoretical basis and technical reference for the development, quality control, and industrial production of functional beverages based on medicine-food homology plant formulas.

## 2. Results and Discussion

### 2.1. Formulation Optimization of P. lobata Compound Beverage

#### 2.1.1. Concentrations of Extract Stock Solution, Mogroside, and Citric Acid

**Figure 1:**
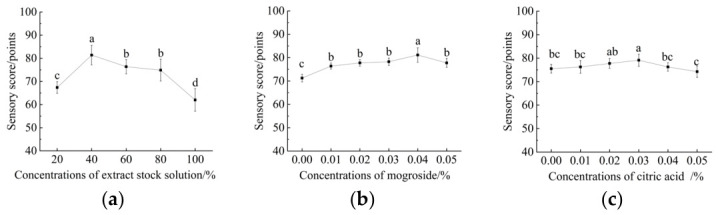
Effect of concentrations of extract stock solution (**a**), mogroside (**b**), and citric acid (**c**) on the sensory score of the *P. lobata* compound beverage. Different lowercase letters indicate significant differences (*p* < 0.05).

Sensory score was used as a primary criterion for preliminary screening and optimization to ensure product acceptability. The sensory quality of the *P. lobata* compound beverage is influenced by the extract stock solution, sweetener, and acidity regulator. As illustrated in Figure 1a, the sensory score of the beverage initially increased and then decreased as the extract stock solution concentration increased. At moderate concentrations, the beverage exhibited a harmonious botanical aroma and balanced taste, whereas excessive addition intensified bitterness and astringency, resulting in a lower sensory score. Therefore, extract stock solution concentrations of 20%, 40%, and 60% (*v*/*v*) were selected for the orthogonal experiment.

As shown in Figure 1b, sensory score peaked at a mogroside concentration of 0.04% (*w*/*v*). At this level, the sweetness effectively neutralized the botanical bitterness and astringency while preventing the lingering unnatural aftertaste associated with excessive sweetener application. Therefore, mogroside concentrations of 0.03%, 0.04%, and 0.05% (*w*/*v*) were selected for the orthogonal experiment.

Citric acid serves as an acidity regulator in beverages. It not only adjusts acidity but also enhances product flavor and aroma. Furthermore, it can inhibit microbial growth [18]. The effect of citric acid concentration on sensory score is shown in Figure 1c. The sensory score reached its maximum when the citric acid concentration was 0.03% (*w*/*v*). If the concentration is too low, the sweet–sour balance of the system is disrupted. Conversely, excessive addition causes a sharp sourness, which impairs the overall taste. Therefore, citric acid concentrations of 0.02%, 0.03%, and 0.04% (*w*/*v*) were selected as the levels for the orthogonal experiment.

Based on the single-factor experiments, the concentrations of the extract stock solution, mogroside, and citric acid were optimized using sensory score as the evaluation index. As shown in Table 1 and Table 2, the influence of each factor on the sensory score followed this order: extract stock solution > mogroside > citric acid. The effects of extract stock solution and mogroside on sensory scores are significant (*p* < 0.05). The optimal formulation combination was determined to be A2B2C1. Specifically, this included 40% (*v*/*v*) extract stock solution, 0.04% (*w*/*v*) mogroside, and 0.02% (*w*/*v*) citric acid. Although the single-factor test showed that sensory acceptance reached its highest value at 0.03% citric acid, this experiment was primarily used to determine the appropriate factor range for subsequent optimization. In the orthogonal experiment, the final formulation was selected based on comprehensive evaluation under multi-factor combination conditions rather than on the single-factor peak alone. Under these conditions, 0.02% citric acid provided a more balanced flavor profile and better compatibility with the other formulation components, and was therefore selected for the final beverage formulation. Three batches of *P*. *lobata* compound beverage were prepared according to this optimal formulation. The average sensory score was 85.40, which was higher than any group in the orthogonal experiment.

#### 2.1.2. Determination of Stabilizer Concentration

**Figure 2:**
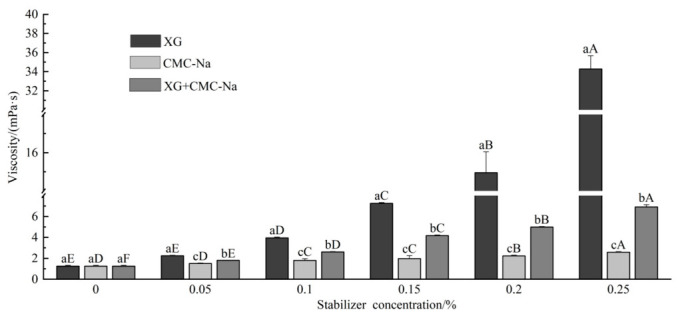
Effect of different stabilizers on the viscosity of *P. lobata* compound beverage. Different uppercase letters indicate significant differences between groups, and different lowercase letters indicate significant differences within groups (*p* < 0.05).

Figure 2 shows the effect of stabilizer concentration on beverage viscosity. Compared with the control group without stabilizer, both XG and CMC-Na significantly increased the system viscosity (*p* < 0.05). This is because both XG and CMC contain hydroxyl (–OH) groups that can bind with water molecules. They interact with water molecules and hydrophilic components, thereby increasing the liquid viscosity [19]. This increase in viscosity is beneficial for improving the physical stability of the beverage system, reducing particle sedimentation and phase separation, but may not be accepted by consumers [20]. At the same concentration, XG exhibited a significantly stronger thickening effect than CMC-Na (*p* < 0.05). This may be attributed to the larger molecular structure, longer chains, and greater branching of XG, which promote hydrogen bond formation and enhance hydration, consequently producing a greater increase in viscosity [19].

Table 3 presents the effects of different stabilizer concentrations on Zeta potential, stability coefficient, and sensory score. The beverage sample without stabilizer had a Zeta potential of −7.17 ± 0.72 mV and a low stability coefficient. This result indicated poor stability. As XG concentration increased, the Zeta potential of the system became more negative. Meanwhile, the stability coefficient increased significantly (*p* < 0.05). However, the higher viscosity reduced fluidity and significantly lowered sensory score (*p* < 0.05). At the same concentration, the Zeta potential of the CMC-Na group did not differ significantly from that of the control group (*p* > 0.05). This may be because the CMC-Na concentration was insufficient to form a dense adsorption layer on the surfaces of colloidal particles [21]. Nevertheless, the stability coefficient increased significantly (*p* < 0.05), likely due to the increased viscosity of the beverage system.

Previous studies have shown that XG and CMC-Na can act synergistically to improve system stability and significantly enhance particle suspension capability and storage stability [8]. Therefore, a 1:1 (*w*/*w*) mixture of XG and CMC-Na was evaluated using the multi-index comprehensive evaluation method. The results indicated that XG alone provided stronger thickening and suspension effects, but its excessive viscosity negatively affected sensory acceptability, whereas CMC-Na alone was more favorable for palatability but showed limited improvement in stability. By contrast, the 1:1 (*w*/*w*) XG and CMC-Na mixture exhibited a significantly higher absolute Zeta potential value than either XG or CMC-Na used alone, indicating improved electrostatic stabilization of the beverage system. The highest comprehensive score was obtained at a total stabilizer concentration of 0.25% (*w*/*v*), reaching 95.64. Therefore, this formulation was selected as the final stabilizer system for the beverage. The beverage prepared with this optimal formulation was then filled and sterilized. The resulting product exhibited a stability coefficient of 21.45 ± 0.44% and a Zeta potential of −16.60 ± 0.26 mV. Notably, the absolute value of this Zeta potential was below the classical empirical criterion commonly associated with electrostatically stabilized colloidal systems (approximately |30| mV) [22]. However, Zeta potential primarily reflects the electrostatic contribution to colloidal stability and therefore cannot fully explain the stability of mixed hydrocolloid systems. For a blended system such as XG and CMC-Na, the observed stability is not determined solely by electrostatic repulsion. Rather, the superior performance of the mixed stabilizer may be attributed to the complementary roles of the two hydrocolloids. Specifically, XG mainly contributes to steric and rheological stabilization by increasing the viscosity of the continuous phase and forming a hydrated three-dimensional network, whereas CMC-Na, as an anionic polysaccharide, may enhance electrostatic repulsion and interfacial stabilization [8]. In addition, intermolecular entanglement between XG and CMC-Na chains may further strengthen the network structure of the continuous phase, thereby improving low-shear stability and reducing particle sedimentation [23].

### 2.2. Volatile Components of the P. lobata Compound Beverage

The volatile flavor compounds in the sample were qualitatively analyzed using GC-MS. As shown in Table 4, a total of 48 volatile compounds were identified. These included 13 aldehydes, 6 alcohols, and 10 terpenes. Small amounts of phenols, alkanes, aromatics, furans, esters, acids, ketones, and ethers were also detected. Aldehydes, accounting for 46.82% of the total volatile content, exhibited the highest relative abundance and were thus the main contributors to the characteristic aroma. Within this group, furfural and nonanal were the predominant compounds. Furfural is typically generated through the Maillard reaction between sugars and amino acids during heating and also results from the decarboxylation of hydroxymethyl furfural [24]. Furfural imparts caramel-like and toasted bread notes to the product. In contrast, straight-chain aliphatic aldehydes, such as nonanal, hexanal, and decanal, are mainly produced by the oxidative degradation of unsaturated fatty acids through the lipoxygenase pathway. These compounds exhibit grassy, citrus, and fatty odor notes [25]. These aliphatic aldehydes are important contributors to the fresh aroma of the sample. In addition, aromatic aldehydes, such as phenylacetaldehyde and benzaldehyde, further enriched the aroma profile.

In this study, volatile components were only determined in the products at day 0, so storage-induced compositional changes could not be assessed. Future studies could perform volatile component analysis at different storage time points to enable direct analysis of the dynamic changes in composition during storage.

### 2.3. Effects of Storage Time and Temperature on Beverage Quality

#### 2.3.1. Changes in pH and SSC During Storage

**Figure 3:**
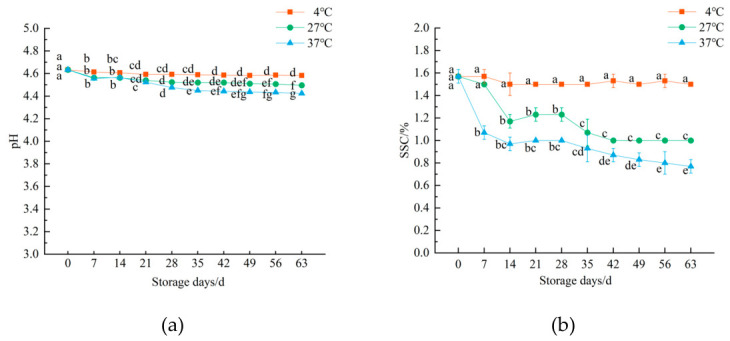
Changes in pH (**a**) and SSC (**b**) of *P. lobata* compound beverage at different storage temperatures. Different lowercase letters indicate significant differences (*p* < 0.05).

Both pH and SSC are important indicators of beverage physicochemical properties. As shown in Figure 3a, the pH of the *P. lobata* compound beverage showed an overall downward trend during storage. The decrease was more pronounced at higher temperatures. At 4 °C, the pH exhibited only marginal fluctuations, with no significant differences observed between days 21 and 63 of storage (*p* > 0.05). Since no microorganisms were detected in the samples throughout the storage period, the observed decrease in pH is more likely attributable to non-microbial chemical reactions. Specifically, the further degradation of sugars and Maillard reaction intermediates during storage may generate small organic acids [26,27]. Such reactions are typically accelerated at higher temperatures, which is consistent with the faster pH decrease observed at 27 °C and 37 °C [28].

The pH decreased more rapidly as storage temperature increased. At 37 °C, pH decreased from 4.63 ± 0.01 to 4.42 ± 0.01. High temperatures likely destabilize the beverage system, accelerating macromolecular degradation and promoting various physicochemical reactions [29].

As shown in Figure 3b, the SSC remained stable at 4 °C (*p* > 0.05) but gradually decreased at 27 °C and 37 °C, indicating superior physical stability under refrigeration. The reduction in SSC may result from the degradation of certain nutrients during storage. Furthermore, elevated temperatures can accelerate the Maillard reaction, thereby converting soluble compounds into insoluble complexes [30]. 

#### 2.3.2. Changes in Color Parameters During Storage

**Figure 4:**
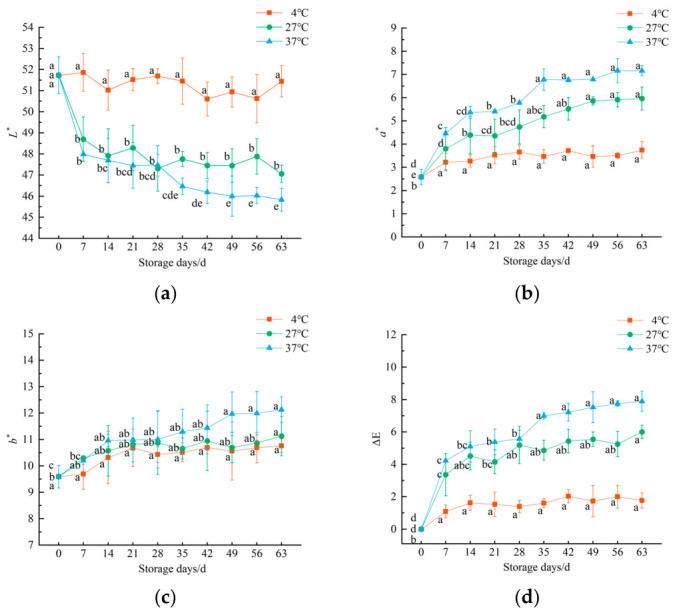
Changes in color parameters L^*^ (**a**), a^*^ (**b**), b^*^ (**c**) and ΔE (**d**) of *P. lobata* compound beverage at different storage temperatures. Different lowercase letters indicate significant differences (*p* < 0.05).

Color is an intuitive indicator of beverage quality for consumers. Figure 4 displays the changes in color parameters during storage. At 4 °C, *L^∗^* and *b^∗^* showed no obvious changes, whereas a∗ and ΔE increased significantly only during the early stage of storage (*p* < 0.05). This suggests that low temperatures help preserve beverage color. However, at 27 °C and 37 °C, *L^∗^* decreased over time, whereas *a^∗^*, *b^∗^*, and ΔE increased. The color change in the beverage may also be influfenced by phenolic constituents derived from *P. lobata*, which is rich in isoflavones and related phenolic compounds, particularly puerarin, daidzin, daidzein, genistin, and genistein [31]. During processing and storage, these compounds may undergo enzymatic browning or non-enzymatic oxidation to form quinone intermediates. These quinones can subsequently participate in nucleophilic addition reactions, electron-transfer reactions with polyphenols, and reactions with amino compounds or proteins, thereby contributing to the formation of brown or reddish-brown chromophoric products [32,33]. Meanwhile, Maillard reaction products, such as melanoidins, formed during storage may further deepen the reddish-brown tone of the system [34].

Storage temperature and time were positively correlated with ΔE. At 4 °C, all ΔE values remained below 3.00. At 37 °C, the ΔE reached 4.23 ± 0.05 after 7 days of storage and increased to 7.89 ± 0.62 after 63 days. Higher temperatures and longer storage periods may increase pigment accumulation, thereby causing pronounced color changes [35]. In general, color differences become readily perceptible when ΔE > 3.5 [29]. Therefore, prolonged exposure to high temperatures should be avoided to reduce color deterioration and maintain sensory acceptance during storage.

#### 2.3.3. Changes in Stability Coefficient and Zeta Potential During Storage

**Figure 5:**
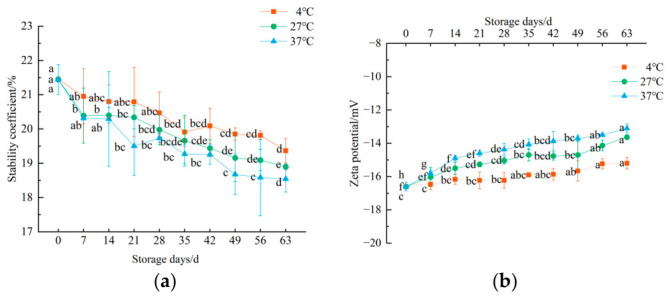
Changes in stability coefficient (**a**) and Zeta potential (**b**) of *P. lobata* compound beverage at different storage temperatures. Different lowercase letters indicate significant differences (*p* < 0.05).

As shown in Figure 5, the physical stability of the beverage gradually deteriorated with prolonged storage, evidenced by a downward trend in both the stability coefficient and the absolute Zeta potential values. These changes were more pronounced at higher temperatures. After 63 days of storage, the Zeta potentials at 4 °C, 27 °C, and 37 °C were −15.20 ± 0.35 mV, −13.63 ± 0.15 mV, and −13.10 ± 0.26 mV, respectively, representing decreases of 8.43%, 17.87%, and 21.08% from initial value. Mechanistically, elevated temperatures weaken the electrostatic repulsion between particles in the system, thereby promoting particle aggregation and sedimentation and ultimately compromising overall physical stability [8].

#### 2.3.4. Changes in Sensory Quality During Storage

Sensory quality is a comprehensive indicator of consumer acceptance. As listed in Table 5 (with the day-0 score defined as 100), sensory score generally decreased over time. However, the rate of decline differed significantly among storage temperatures. At 4 °C, sensory changes were minor, and the initial flavor characteristics were well preserved. At 27 °C, the score gradually declined but remained acceptable for a certain period. At 37 °C, the score experienced the most rapid decline, dropping to 57.75 ± 4.59 after 63 days of storage. This deterioration was characterized by a darker color, weaker aroma, poorer physical stability, and inferior taste. Active components such as polyphenols may undergo oxidative degradation during storage, accompanied by enzymatic and non-enzymatic browning reactions, thereby deepening beverage color and reducing brightness [32,33]. Meanwhile, fine *P. lobata* starch granules and other plant-derived insoluble particles present in the beverage may gradually settle under gravity or aggregate into larger flocs, accelerating sedimentation and adversely affecting beverage stability [36,37]. In addition, oxidation and polymerization of phenolic compounds during storage may enhance their affinity for proteins and salivary proteins, disrupt the salivary lubricating film, increase oral friction, reduce smoothness, and consequently intensify dryness and astringency [38,39]. Furthermore, volatile aroma compounds may be gradually lost or transformed during storage through volatilization and oxidation, resulting in diminished aroma intensity or the development of off-flavor notes [40]. Ultimately, these changes reduce the coordination of appearance, aroma, and taste, leading to a lower sensory score. These sensory results were consistent with the aforementioned changes in color parameters and physical stability indicators.

**Figure 6:**
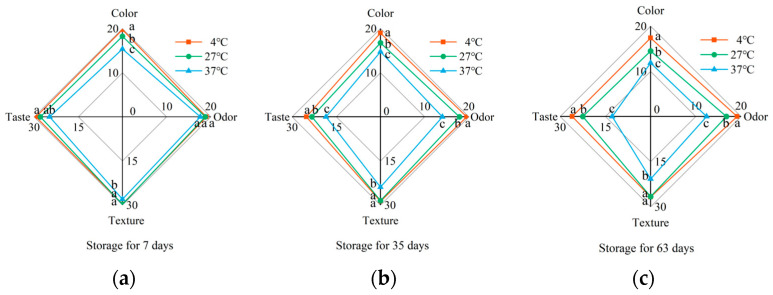
Radar chart of sensory evaluation of *P. lobata* compound beverage stored for 7 d (**a**), 35 d (**b**), and 63 d (**c**) at different storage temperatures. Different lowercase letters indicate significant differences (*p* < 0.05).

Figure 6 presents a radar chart illustrating the sensory attributes on days 7, 35, and 63 of storage. The taste score exhibited the most pronounced decline, characterized by reduced flavor harmony and increased astringency. The color changes were mainly attributed to non-enzymatic browning, which encompasses caramelization, the Maillard reaction, vitamin C degradation, and the oxidative polymerization of phenols [41]. Furthermore, the changes in texture were closely associated with particle aggregation and sedimentation.

#### 2.3.5. Changes in Active Components During Storage

**Figure 7:**
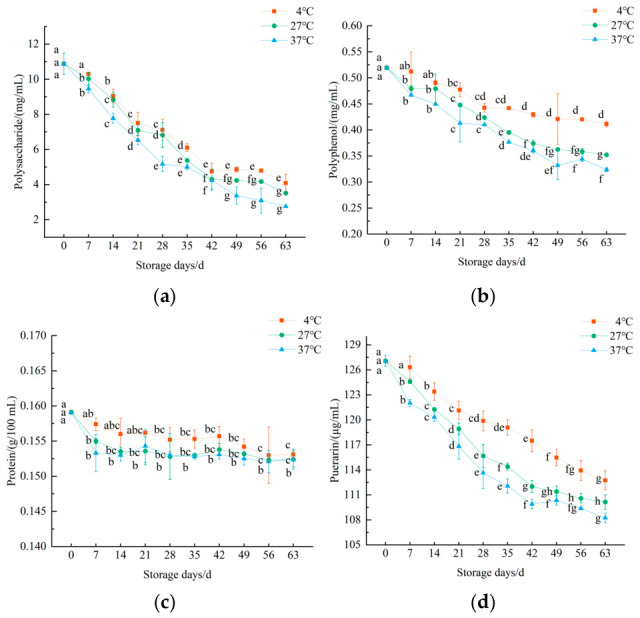
Changes in the polysaccharide (**a**), polyphenol (**b**), protein (**c**), and puerarin (**d**) of *P. lobata* compound beverage at different storage temperatures. Different lowercase letters indicate significant differences (*p* < 0.05).

As illustrated in Figure 7, the contents of all active components exhibited a downward trend over time. Higher temperatures led to faster degradation rates. After 63 days at 37 °C, polysaccharide content decreased by 74.69% compared with day 0. This indicates that polysaccharide was the most temperature-sensitive active components in this system. Polysaccharide may undergo thermal-induced depolymerization during storage, leading to cleavage of glycosidic bonds and conversion of high-molecular-weight polysaccharides into lower-molecular-weight fragments or oligosaccharides [42]. In addition, in the beverage system, polysaccharide may interact with proteins and phenolic compounds through non-covalent or covalent associations, which can promote particle aggregation and precipitation, thereby reducing the soluble polysaccharide content [43]. Consequently, polyphenol content also decreased significantly. Moreover, elevated temperatures further accelerate such interactions between polyphenols and proteins [44]. In contrast, the protein content remained relatively stable, decreasing by only 4.21% after 63 days at 37 °C. Mao et al. [45] reported that the addition of XG and CMC-Na to protein-rich aqueous solutions effectively maintains protein structural stability. This hydrocolloid combination helps preserve protein content after thermal processing and storage. Furthermore, puerarin decreased by only 14.83% after 63 days at 37 °C, indicating its excellent storage stability in this beverage. As a representative isoflavone in *P*. *lobata*, puerarin has a relatively stable chemical structure. Li et al. [46] simulated the storage of Pueraria-Ophiopogon tea and found that puerarin retained more than 90% of its initial concentration after 3 months at 37 °C and 75% relative humidity.

To further clarify these behaviors, degradation kinetics were analyzed. As shown in Table 6, the degradation of polysaccharide, polyphenol, and puerarin fitted the first-order kinetic model well. This indicates that their degradation rates depended mainly on their current concentrations, with the degradation rate constant increasing with storage temperature. Therefore, temperature is a crucial factor affecting their stability. However, the minimal changes in protein content during storage prevented it from fitting either the zero-order or first-order kinetic models.

### 2.4. Shelf-Life Prediction

**Figure 8:**
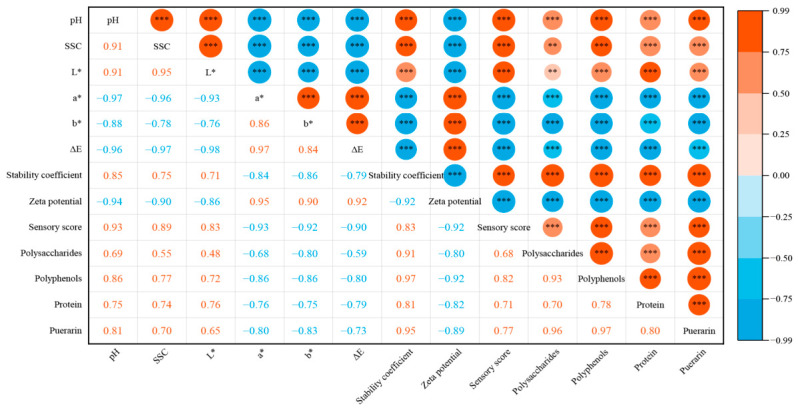
Pearson correlation analysis plot between sensory score and various physicochemical indicators. ** *p* ≤ 0.01 *** *p* ≤ 0.001.

In shelf-life evaluation, microbiological indicators are typically used to assess safety, whereas sensory and physicochemical parameters reflect quality changes. In this study, the three microbiological indicators, namely total viable count, coliforms, and molds/yeasts, were examined at all storage time points, and no microorganisms were detected in any sample throughout storage. This result indicates that the sterilization treatment used in this study was effective under the tested conditions. Therefore, microbiological indicators could not be used to define the shelf-life endpoint. A Pearson correlation analysis was conducted to identify suitable prediction parameters (Figure 8). Correlations varied markedly among the different indicators. Specifically, pH, a^*^, b^*^, ΔE and Zeta potential were strongly correlated with sensory score, with their correlation coefficients all exceeding 0.90.

The changes in pH, a^*^, b^*^, and ΔE failed to fit either zero- or first-order kinetics. Therefore, sensory score and the absolute value of Zeta potential were selected as the key indicators for shelf-life prediction. This choice is further supported by a plausible mechanistic relationship between particle stability and sensory perception. Specifically, a decrease in the absolute value of Zeta potential indicates weakened electrostatic repulsion between dispersed particles, thereby promoting aggregation, flocculation, and sedimentation [22]. Such aggregation behavior may reduce dispersion homogeneity and impair oral lubrication, thereby contributing to undesirable sensory attributes such as roughness, grittiness, reduced smoothness, while enhanced dryness and astringency [40,47]. Carter et al., working with whey protein systems, directly confirmed that the closer the Zeta potential is to zero, the higher the astringency intensity [48]. Therefore, reduced particle stability directly intensifies astringency perception and degrades mouthfeel texture by altering physical friction and lubrication. The fitted models are presented in Table 7.

The shelf-life of the beverage was then predicted using linear regression equations and the Arrhenius equation. According to the sensory evaluation standard adopted in this study, a total sensory score of 60 was defined as the minimum acceptable limit for samples. When the total sensory score of a sample fell below 60, the panelists generally considered that the sample exhibited obvious defects in one or more attributes, including color, odor, texture, or taste. Therefore, a score of 60 was used as the threshold for shelf-life determination. According to the regression equation between sensory score and Zeta potential (sensory score = −10.28 × Zeta potential − 70.298, R^2^ = 0.8538), the critical value of Zeta potential is calculated to be –12.67 mV. The coefficients of determination (R^2^) for the Arrhenius fitting of sensory score and Zeta potential were 0.8486 and 1.0000, respectively, indicating that the Arrhenius equation adequately describes the temperature dependence of degradation and supports subsequent shelf-life prediction. The corresponding Arrhenius plots are shown in Figure S1. The shelf-life prediction results are shown in Table 8. Based on sensory score, the predicted shelf lives at 4 °C, 27 °C, and 37 °C were 309, 125, and 88 days, respectively. Based on Zeta potential predictions, the shelf lives at 4 °C, 27 °C, and 37 °C were 193, 104, and 82 days, respectively. Sensory score reflects the overall color, aroma, taste, and overall acceptability, making it a comprehensive quality indicator. In contrast, Zeta potential more directly reflects the charge stability of the dispersed system and is more sensitive to early physical instability events such as particle aggregation, sedimentation, and phase separation. Therefore, Zeta potential tends to give a more conservative and shorter shelf-life prediction, while sensory score only shows a significant decline when these changes have further developed to a level that is clearly perceptible by human senses. Compared with some other plant-based beverages, this product exhibits a longer shelf life [49,50].

## 3. Materials and Methods

### 3.1. Materials and Reagents

Raw plant materials, including *P. lobata*, *G. lucidum*, *L. barbarum*, and *H. dulcis*, were purchased from Beijing Tongrentang Nanjing Pharmacy Co., Ltd. (Nanjing, China). Food-grade mogroside, citric acid, XG, and CMC-Na were supplied by Jiahe Food Industry Co., Ltd. (Suzhou, China). An analytical standard of puerarin (purity ≥ 98% by HPLC) was obtained from Shanghai Yuanye Bio-Technology Co., Ltd. (Shanghai, China). Chromatographic-grade acetonitrile was purchased from Sinopharm Wokai Chemical Reagent Co., Ltd. (Shanghai, China). All other chemical reagents, including phenol, sulfuric acid, Folin–Ciocalteu reagent, sodium carbonate, copper sulfate, and potassium sulfate, were of analytical grade and provided by Sinopharm Chemical Reagent Co., Ltd. (Shanghai, China).

### 3.2. Preparation of Extract Stock Solution

Dried plant materials were crushed prior to extraction. Specific amounts were weighed: 1.40 g of *P. lobata*, 12.00 g of *G. lucidum*, 4.00 g of *L. barbarum*, and 13.60 g of *H. dulcis*. This dosage ratio was determined based on preliminary experiments and formulation optimization previously conducted in our laboratory, the detailed experimental design is provided in the Supplementary Material. These materials were mixed and soaked in hot water (1:10, *w*/*v*) for 30 min. The mixture was then reflux-extracted in boiling water for 1 h under normal pressure. After extraction, the mixture was filtered, and the filtrate was collected. The remaining residue underwent a second extraction with boiling water (1:10, *w*/*v*) for 1 h. This mixture was also filtered. Filtrates from both extractions were combined. The combined filtrate was centrifuged at 5000 rpm for 30 min using a DL-5 B low-speed centrifuge (Anting Scientific Instrument Co., Ltd., Shanghai, China). The supernatant was then concentrated under reduced pressure at 50 °C to one-quarter of its original volume. The concentrated volume was 155 mL, and the resulting product was the compound extract stock solution.

### 3.3. Formulation Optimization of P. lobata Compound Beverage

#### 3.3.1. Single-Factor Experiments

Single-factor experiments were conducted using sensory score as the evaluation index. During the evaluation of a single factor, the other two variables remained constant. The baseline formulation included 60% (*v*/*v*) extract stock solution, 0.03% (*w*/*v*) mogroside, and 0.03% (*w*/*v*) citric acid. Specifically, when evaluating the extract stock solution, the concentrations of mogroside and citric acid were fixed at 0.03% (*w*/*v*) each. The extract stock solution (20, 40, 60, 80,100%, *v*/*v*), mogroside (0.01, 0.02, 0.03, 0.04, 0.05%, *w*/*v*), and citric acid (0.01, 0.02, 0.03, 0.04, 0.05%, *w*/*v*) were systematically evaluated across five concentration gradients, respectively.

#### 3.3.2. Orthogonal Optimization Experiment

A three-factor, three-level orthogonal experiment was designed based on the single-factor results. The sensory score was used as the evaluation index to optimize the formulation. The factors included the concentrations of the extract stock solution (A), mogroside (B), and citric acid (C). The factors and levels are shown in Table 9.

#### 3.3.3. Determination of Stabilizer Concentration

XG and CMC-Na were selected as stabilizers. Their stabilizing effects on the beverage were evaluated individually and as a mixture (1:1, *w*/*w*). Five concentration levels (0.05%, 0.10%, 0.15%, 0.20%, and 0.25%, *w*/*v*) of these stabilizers were added to the formulated beverage. Centrifugal precipitation rate, Zeta potential, and sensory score were measured.

The multi-index comprehensive evaluation method was used to determine the type and concentration of stabilizers [51]. The steps were as follows:

(1) Score conversion

The optimal measured value (*x_b_*) among the indicators was set to a full score of 100. The remaining measured values were converted into relative scores (*X_ij_*).

For positive indicators, the score was calculated using Equation (1):

(1) $$X_{i\;j}\;=\;\frac{{100\;\times \;x}_{\mathrm{ij}}}{x_{b}}$$

For negative indicators, the score was calculated using Equation (2):

(2) $$X_{i\;j}\;=\;\frac{{100\;\times \;x}_{b}}{x_{\mathrm{ij}}}$$

where *X_ij_* is the converted score of the j-th indicator in the i-th group (maximum 100); *x_ij_* is the actual measured value; and *x_b_* is the optimal actual measured value among all groups.

(2) Objective weighting by the coefficient of variation method

Objective weights for each indicator were calculated using Equation (3):

(3) $$W_{j}\;=\;\frac{V_{j}}{\sum_{j=1}^{n}V_{j}}$$

where *W_j_* is the weight coefficient of the j-th indicator; *V_j_* is the coefficient of variation for the j-th indicator; and n is the total number of indicators.

(3) Calculation of comprehensive score

The final comprehensive score (*P_i_*) was calculated using Equation (4):

(4) $$P_{i}=\Sigma_{j=1}^{n}\left(X_{\mathrm{ij}}{\;\times \;W}_{j}\right)$$

where *P_i_* is the multi-index comprehensive score for the i-th test group.

### 3.4. Preparation of P. lobata Compound Beverage Samples

The beverage was prepared based on the optimized formulation detailed in Section 3.3. Water was added to the extract stock solution to achieve the target volume. Mogroside, citric acid, and stabilizers were subsequently added. The solution was thoroughly mixed. The mixture was homogenized twice at 25 MPa using a homogenizer. Subsequently, 50 mL aliquots were dispensed into light-resistant brown glass bottles and sealed. The sealed bottles were sterilized at 121 °C for 15 min. The final *P. lobata* compound beverage was obtained after cooling to room temperature. The samples were stored at 4 °C, 27 °C, and 37 °C for 63 days. Samples were taken every 7 days to measure quality changes.

### 3.5. Determination of Volatile Components

The volatile components of the samples that had been sterilized and cooled to room temperature, namely the samples stored for 0 days, were analyzed using a TSQ 8000 triple quadrupole gas chromatography-mass spectrometry (GC-MS) system (Thermo Fisher Scientific, Waltham, MA, USA). Static headspace sampling was employed for volatile extraction, with incubation at 55 °C for 40 min. Chromatographic separation was performed on a DB-WAX capillary column (30 m × 0.25 mm, 0.25 μm), using helium as the carrier gas at a flow rate of 1.000 mL/min. The inlet temperature was 260 °C, and the injection volume was 1.0 μL. The temperature program was set as follows: initial temperature of 40 °C (held for 2 min); increased to 200 °C at 3 °C/min; and further increased to 250 °C at 10 °C/min (held for 5 min). A splitless injection mode was employed. Mass spectrometry utilized an electron ionization (EI) source. The ion source temperature was 300 °C, and the transfer line temperature was 280 °C. The electron impact energy was 70 eV. Data were acquired in Full Scan mode with a mass range of *m*/*z* 33–400. The resulting mass spectra of chromatographic peaks were matched against the NIST standard mass spectral library for compound identification. Relative contents of volatile components were calculated using the peak area normalization method.

### 3.6. Physicochemical Analysis of P. lobata Compound Beverage During Storage

#### 3.6.1. pH and SSC

The pH value was measured using an ST3100 pH meter (Ohaus Instruments Co., Ltd., Changzhou, China) according to the National Food Safety Standard (GB 5009.237–2016) [52]. The soluble solid content (SSC) was measured using a refractometer, following the General Methods for Beverage Analysis (GB/T 12143-2008) [53].

#### 3.6.2. Color Parameters

Color parameters were evaluated using a DS-200 colorimeter (Caipu Technology Co., Ltd., Hangzhou, China) based on the CIELAB color space. The lightness (L^*^), redness/greenness (a^*^), and yellowness/blueness (b^*^) were recorded. The total color difference (ΔE) was calculated using Equation (5).

(5) $$\Delta E=\sqrt{{\left(L^{*}{-L}_{0}^{*}\right)}^{2}+{\left(a^{*}{-a}_{0}^{*}\right)}^{2}+{\left(b^{*}{-b}_{0}^{*}\right)}^{2}}$$

where L^*^, a^*^ and b^*^ are the color values of the stored sample; and L^*^_0_, a^*^_0_, and b^*^_0_ are the baseline color values of the sample at day 0.

#### 3.6.3. Stability Coefficient

The stability coefficient was determined following a previously described method [54]. The absorbance was measured using a UV-1600 UV-Vis spectrophotometer (Mapada Instruments Co., Ltd., Shanghai, China) before and after centrifugation. The stability coefficient was calculated using Equation (6).

Stability coefficient (%) = (Absorbance of supernatant after centrifugation/Absorbance of sample before centrifugation) × 100%(6)

#### 3.6.4. Zeta Potential

Samples were diluted with deionized water at a ratio of 1:10 (*v*/*v*) and mixed evenly. The dispersion was transferred to a Malvern cuvette. The Zeta potential was measured using a Nano-ZSE analyzer (Malvern Instruments Ltd., Worcestershire, UK).

#### 3.6.5. Active Components

Polysaccharide and polyphenol contents were determined following the previously described method with slight modifications [4]. Protein content was measured using the Kjeldahl method (GB 5009.5-2025) [55]. The content of puerarin was determined using a QuikSep analytical and semi-preparative HPLC system (Beijing HuiDeXin Technology Co., Ltd., Beijing, China) equipped with an Agilent C18 column (4.6 × 150 mm, 5 μm). The injection volume was 10 μL, and all other conditions were in accordance with the method specified in GB/T 22251-2024 [56]. Quantification was performed using the external standard method, and a calibration curve was constructed by plotting the peak areas of the standards against their concentrations to calculate the puerarin content in the samples.

#### 3.6.6. Microbial Indicators

Microbial parameters were assessed according to National Food Safety Standards. The total viable count was measured according to GB 4789.2-2022 [57]. Coliforms were enumerated according to GB 4789.3-2025 [58]. Molds and yeasts were counted according to GB 4789.15-2016 [59].

### 3.7. Sensory Evaluation

The sensory evaluation of the *P. lobata* compound beverage samples was conducted by the Center for Food Nutrition and Functional Factors at Jiangnan University (Wuxi, China). This protocol has been approved by the Medical Ethics Committee of Jiangnan University (Reference No.: JNU202509RB058). The specific experimental protocol is as follows [60,61]. A 10-member panel was formed, consisting of students from Jiangnan University (5 females and 5 males, aged 20 to 25), all of whom were familiar with descriptive analysis procedures. The panelists had prior experience in sensory analysis of beverage products and had no food intolerances or allergies, and they signed informed consent before the test. First, the panelists described the sensory attributes related to the product’s color, odor, texture, and taste. Through group consensus, ambiguous or redundant terms were removed. Subsequently, the panel defined scale intensity for each identified sensory attribute and used appropriate descriptive terms. Finally, a subset of samples was used to train the panel to become familiar with the product characteristics. In this study, the panelists tested different formulations of the *P. lobata* compound beverage, with samples labeled using three-digit random numbers. Six to nine samples were tested at a time, and the evaluation was repeated twice. A two-minute rest is provided before each tasting to avoid sensory fatigue. After each tasting, panelists should cleanse their palates with water and soda crackers, and complete a questionnaire to evaluate color, odor, texture, and taste. Each panelist evaluated the samples independently, with no communication between them. Before each assessment, the panelists took a two-minute break to avoid fatigue. The scoring standards are detailed in Table 10.

### 3.8. Shelf-Life Prediction

The shelf-life was predicted following the previously described method [10]. A shelf-life prediction model was established by combining zero-order kinetics, first-order kinetics, and the Arrhenius equation. Equations are given in Equations (7)–(9).

(7) $$\mathrm{Zero}\text{-}\mathrm{order}\;\mathrm{kinetic}\;\mathrm{model}:\;A=A_{0}-K_{a}t$$

(8) $$\mathrm{First}\text{-}\mathrm{order}\;\mathrm{kinetic}\;\mathrm{model}:{\;A=A}_{0}e^{-K_{a}t}$$

(9) $$\mathrm{Arrhenius}\;\mathrm{equation}:\;K_{a}{=K}_{a0}e^{\frac{-E_{a}}{RT}}$$

where A is the quality index value at any time point; t is the storage days (d); *K_a_* is the reaction rate constant; *K_a_*_0_ is the pre-exponential factor; *T* is the absolute storage temperature (K); *E_a_* is the activation energy of the reaction (J/mol); and R is the universal gas constant (8.314 J/(mol·K)).

### 3.9. Data Analysis

Data were analyzed by a one-way analysis of variance (ANOVA) followed by Duncan’s multiple comparison using SPSS 16.0 software (IBM Corp., Armonk, NY, USA). The results are expressed as mean ± standard deviation. Graphs were plotted using Origin 2026 software (OriginLab Corp., Northampton, MA, USA). Different lowercase letters in the figures indicate significant differences between groups (*p* < 0.05). All experiments were repeated in triplicate.

## 4. Conclusions

This study systematically investigated the *P. lobata* compound beverage, encompassing formulation optimization, stabilizer screening, volatile profiling, storage quality dynamics, and shelf-life prediction. The optimal formulation consisted of 40% (*v*/*v*) extract stock solution, 0.04% (*w*/*v*) mogroside, 0.02% (*w*/*v*) citric acid and 0.25% (*w*/*v*) composite stabilizer (XG and CMC-Na). This optimized system effectively improved the taste and enhanced physical stability. GC-MS analysis identified multiple volatile compounds that collectively formed the characteristic flavor profile. During storage, physicochemical properties, sensory quality, and active component contents exhibited varying degrees of alteration, with higher temperatures significantly accelerating these deterioration processes. Zeta potential showed a strong correlation with sensory score and fitted the first-order kinetic model well, indicating its suitability as a predictive indicator for shelf-life. Based on Zeta potential, the predicted shelf lives at 4 °C, 27 °C, and 37 °C were 193, 104, and 82 days, respectively. These findings provide a sound theoretical basis for formulation design, stability improvement, and shelf-life evaluation of functional *P. lobata* beverages. Considering the impact of the sterilization conditions used in this experiment on the active components and stability of the beverage, future work could further compare milder or alternative sterilization processes to better balance microbial safety and quality preservation. Furthermore, the critical Zeta potential value was indirectly derived from sensory score, a subjective indicator, through regression analysis. Therefore, although Zeta potential serves as an effective predictor with mechanistic relevance and good kinetic fitting, the critical value still depends on sensory evaluation and has not yet been independently validated. Further studies are needed to confirm the robustness of this critical value using independent validation approaches.

## Figures and Tables

**Table 1:** Analysis of range for orthogonal experimental design results.

No.	A (Concentrations of Extract Stock Solution)/%	B (Concentrations of Mogroside)/%	C (Concentrations of Citric Acid)/%	Sensory Score
1	1	1	1	77.40
2	1	2	2	80.20
3	1	3	3	80.00
4	2	1	2	80.30
5	2	2	3	84.90
6	2	3	1	83.90
7	3	1	3	72.30
8	3	2	1	76.70
9	3	3	2	77.30
M1	79.20	76.67	79.33	
M2	83.03	80.60	79.27	
M3	75.43	80.40	79.07	
R	7.60	3.93	0.27	

Note: M1 denotes the average sensory score at level 1, M2 denotes the average sensory score at level 2, and M3 denotes the average sensory score at level 3. R represents the range, and R = max(M1, M2, M3) − min(M1, M2, M3).

**Table 2:** Analysis of variance results for orthogonal experimental design.

Factor	Sum of Squares	df	F-Value	*p*-Value	Significance
A	866.42	2	39.71	<0.05	Significant
B	294.49	2	13.5	<0.05	Significant
C	1.16	2	0.053	>0.05	Not significant

**Table 3:** Results of the multi-indicator empirical formula analysis.

No.	XG Concentration (%)	CMC-Na Concentration (%)	Zeta Potential (mV)	Stability Coefficient (%)	Sensory Score (Points)	Comprehensive Score
1	0.000	0.000	−7.17 ± 0.72 ^a^ (22.97)	29.60 ± 0.34 ^h^ (47.80)	84.10 ± 3.31 ^a^ (100.00)	35.29
2	0.050	0.000	−10.45 ± 1.35 ^a^ (33.45)	46.55 ± 2.38 ^ef^ (75.18)	77.30 ± 5.46 ^bcd^ (91.91)	47.78
3	0.100	0.000	−21.47 ± 1.33 ^c^ (68.73)	49.67 ± 2.26 ^de^ (80.21)	74.30 ± 6.17 ^d^ (88.35)	72.99
4	0.150	0.000	−23.27 ± 1.60 ^cd^ (74.49)	54.43 ± 1.71 ^c^ (87.90)	69.30 ± 4.30 ^e^ (82.40)	78.12
5	0.200	0.000	−28.00 ± 2.44 ^efg^ (89.65)	58.33 ± 1.51 ^b^ (94.20)	69.10 ± 5.38 ^e^ (82.16)	89.96
6	0.250	0.000	−26.03 ± 2.71 ^def^ (83.35)	61.92 ± 1.37 ^a^ (100.00)	67.00 ± 4.97 ^e^ (79.67)	86.64
7	0.000	0.050	−10.63 ± 0.92 ^a^ (34.04)	46.25 ± 1.25 ^f^ (74.69)	80.70 ± 3.80 ^abc^ (95.96)	48.45
8	0.000	0.100	−8.69 ± 1.84 ^a^ (27.81)	50.79 ± 0.39 ^de^ (82.03)	77.80 ± 3.26 ^bcd^ (92.51)	45.41
9	0.000	0.150	−9.71 ± 0.59 ^a^ (31.10)	48.86 ± 0.40 ^def^ (78.90)	78.50 ± 4.30 ^bcd^ (93.34)	47.09
10	0.000	0.200	−9.22 ± 0.33 ^a^ (29.51)	49.55 ± 3.47 ^def^ (80.02)	81.00 ± 3.30 ^abc^ (96.31)	46.49
11	0.000	0.250	−8.87 ± 0.75 ^a^ (28.39)	50.88 ± 0.38 ^d^ (82.16)	80.30 ± 2.71 ^abc^ (95.48)	46.11
12	0.025	0.025	−16.43 ± 3.35 ^b^ (52.61)	40.44 ± 0.55 ^g^ (65.30)	81.70 ± 4.24 ^ab^ (97.15)	59.38
13	0.050	0.050	−24.37 ± 2.00 ^cde^ (78.01)	41.31 ± 2.35 ^g^ (66.71)	79.00 ± 3.68 ^bcd^ (93.94)	76.99
14	0.075	0.075	−28.07 ± 4.70 ^efg^ (89.86)	48.53 ± 0.80 ^def^ (78.37)	78.90 ± 5.20 ^bcd^ (93.82)	87.72
15	0.100	0.100	−28.47 ± 1.90 ^fg^ (91.14)	51.42 ± 3.38 ^cd^ (83.03)	77.40 ± 5.15 ^bcd^ (92.03)	89.46
16	0.125	0.125	−31.23 ± 1.40 ^g^ (100.00)	51.88 ± 1.45 ^cd^ (83.79)	76.30 ± 6.93 ^cd^ (90.73)	95.64
Weighting Coefficient			0.69	0.22	0.09	

Different lowercase letters in the same column indicate significant differences (*p* < 0.05).

**Table 4:** Volatile components of *P. lobata* compound beverage.

No.	Category	Name	CAS	Relative Content (%)
1	Aldehydes	Furfural	98-01-1	15.65 ± 2.74
2		Nonanal	124-19-6	8.85 ± 1.00
3		3,4-Dimethylbenzaldehyde	5973-71-7	5.28 ± 0.85
4		Hexanal	66-25-1	3.28 ± 0.69
5		3-Methylbutanal	590-86-3	2.67 ± 0.52
6		Phenylacetaldehyde	122-78-1	2.62 ± 0.29
7		Decanal	112-31-2	2.38 ± 0.53
8		Benzaldehyde	100-52-7	1.61 ± 0.36
9		Dodecanal	112-54-9	1.29 ± 0.54
10		Octanal	124-13-0	1.01 ± 0.10
11		(E)-2-Nonenal	18829-56-6	0.94 ± 0.16
12		2-Methylbutanal	96-17-3	0.89 ± 0.17
13		Undecanal	112-44-7	0.35 ± 0.07
14	Alcohols	Decyl alcohol	112-30-1	13.50 ± 10.42
15		Linalool	78-70-6	2.11 ± 0.21
16		2-Ethylhexanol	104-76-7	1.70 ± 0.38
17		Alpha-Terpineol	98-55-5	1.09 ± 0.21
18		Bicyclo [2.2.1]heptan-2-ol, 5,5-dimethyl-6-methylene-	3570-04-5	0.52 ± 0.06
19		Cedrol	77-53-2	0.44 ± 0.07
20	Alkenes	Cedrene isomer	22567-43-7	10.00 ± 1.20
21		Terpinolene	586-62-9	1.62 ± 0.33
22		Cycloheptatriene	544-25-2	1.45 ± 0.31
23		2-Bornene	464-17-5	1.41 ± 0.27
24		(S)-(-)-Limonene	5989-54-8	1.26 ± 0.14
25		(+)-β-Funebrene	79120-98-2	1.03 ± 0.07
26		(-)-α-Cedrene	469-61-4	0.50 ± 0.07
27		α-Pinene	80-56-8	0.49 ± 0.28
28		Ocimene mixture of isomers	3338-55-4	0.35 ± 0.08
29		1,5,5-Trimethyl-6-methylenecyclohexene	514-95-4	0.22 ± 0.03
30	Phenols	Phenol	108-95-2	4.04 ± 0.87
31	Alkanes	N-Nonadecane	629-92-5	1.05 ± 0.21
32		Heptadecane	629-78-7	1.04 ± 0.05
33		2,6,10-Trimethyltetradecane	14905-56-7	0.62 ± 0.66
34	Aromatics	P-Xylene	106-42-3	0.59 ± 0.20
35		O-Cymene	527-84-4	0.55 ± 0.09
36		1,1,6-Trimethyltetralin	475-03-6	0.54 ± 0.36
37		Cadalene	483-78-3	0.29 ± 0.04
38		1,5,8-Trimethyl-1,2-dihydronaphthalene	4506-36-9	0.26 ± 0.06
39	Furans	2-Pentylfuran	3777-69-3	1.90 ± 0.39
40		2-Ethylfuran	3208-16-0	0.26 ± 0.05
41	Esters	Octyl acetate	112-14-1	0.91 ± 0.32
42		Ethyl acetate	141-78-6	0.38 ± 0.14
43		Methyl arachidonate	2566-89-4	0.35 ± 0.10
44	Acids	Acetic acid	64-19-7	1.28 ± 0.23
45	Ketones	6-Methyl-5-hepten-2-one	110-93-0	0.49 ± 0.11
46		Geranylacetone	3796-70-1	0.23 ± 0.06
47		4-Methyl-2-pentanone	108-10-1	0.22 ± 0.03
48	Ethers	1,8-Cineole	470-82-6	0.46 ± 0.08

**Table 5:** Changes in sensory score of *P. lobata* compound beverage during storage.

Storage Days/d	Sensory Score/Points		
	4 °C	27 °C	37 °C
0	100.00 ± 0.00 ^a^	100.00 ± 0.00 ^a^	100.00 ± 0.00 ^a^
7	97.75 ± 1.83 ^ab^	94.63 ± 1.77 ^b^	85.75 ± 4.27 ^b^
14	96.75 ± 2.25 ^bc^	90.13 ± 3.68 ^c^	80.63 ± 3.16 ^c^
21	94.13 ± 2.36 ^cd^	89.38 ± 2.07 ^c^	75.13 ± 0.99 ^d^
28	94.13 ± 1.89 ^cd^	88.63 ± 3.93 ^cd^	73.75 ± 3.01 ^de^
35	92.38 ± 4.07 ^d^	86.38 ± 1.77 ^de^	71.13 ± 2.03 ^ef^
42	91.63 ± 3.42 ^de^	85.38 ± 1.30 ^e^	70.25 ± 2.49 ^f^
49	91.38 ± 3.07 ^de^	84.25 ± 1.75 ^ef^	67.25 ± 4.06 ^g^
56	89.00 ± 2.07 ^e^	82.63 ± 1.19 ^fg^	62.38 ± 1.41 ^h^
63	89.25 ± 1.83 ^e^	80.38 ± 2.39 ^g^	57.75 ± 4.59 ^i^

Different lowercase letters in the same column indicate significant differences (*p* < 0.05).

**Table 6:** Parameters of degradation kinetic models for active components of *P. lobata* compound beverage during storage.

Active Components	Storage Temperature (°C)	Zero-Order Kinetic Model		First-Order Kinetic Model		Model Selection
		Ka	R^2^	Ka	R^2^	
Polysaccharide	4	0.1124	0.9442	0.0163	0.9657	First-order
	27	0.1212	0.9428	0.0188	0.9707	
	37	0.1271	0.9381	0.0233	0.9907	
Polyphenol	4	0.0018	0.9347	0.0040	0.9434	First-order
	27	0.0028	0.9572	0.0065	0.9674	
	37	0.0029	0.9434	0.0073	0.9612	
Puerarin	4	0.2312	0.9879	0.0019	0.9898	First-order
	27	0.2787	0.9437	0.0024	0.9503	
	37	0.2861	0.9126	0.0025	0.9218	

**Table 7:** Parameters of degradation kinetic models for active components of *P. lobata* compound beverage during storage.

Index	Storage Temperature/°C	Zero-Order Kinetic Model		First-Order Kinetic Model		Model Selection
		Ka	R^2^	Ka	R^2^	
Sensory score	4	0.1681	0.9590	0.0018	0.9628	First-order
	27	0.2634	0.9197	0.0030	0.9348	
	37	0.5437	0.9021	0.0072	0.9362	
Zeta potential	4	0.0218	0.9306	0.0014	0.9269	First-order
	27	0.0397	0.9375	0.0026	0.9404	
	37	0.0486	0.9125	0.0033	0.9300	

**Table 8:** Shelf-life prediction for *P. lobata* compound beverage.

Index	Storage Temperature/°C	E_a_/(J/mol)	K_a0_	Predicted Shelf Lives/d
Sensory score	4	27,110.29	212.72	309
	27			125
	37			88
Zeta potential	4	18,578.46	4.44	193
	27			104
	37			82

**Table 9:** Factors and levels of orthogonal experiment.

Levels	Factors		
	A (%)	B (%)	C (%)
1	20	0.03	0.02
2	40	0.04	0.03
3	60	0.05	0.04

**Table 10:** Sensory evaluation standards of the *P. lobata* compound beverage.

Index	Evaluation Standard	Sensory Score
Color	Yellowish-brown, bright color, uniform distribution.	15–20
	Yellowish-brown, relatively bright, slightly uneven color distribution	10–14
	Yellowish-brown, dull, too dark or too light, uneven color distribution	0–9
Odor	Harmonious aroma, characteristic fresh scent of *P. lobata*, no off-odor.	15–20
	Acceptable aroma, detectable *P. lobata* scent, no off-odor.	10–14
	Weak *P. lobata* scent, obvious off-odor.	0–9
Texture	Uniform and stable solution, no precipitation or stratification.	20–30
	Relatively uniform solution, slight precipitation.	10–19
	Abundant precipitation, severe stratification.	0–9
Taste	Excellent mouthfeel, proper sweet–sour balance, distinct *P. lobata* flavor, no astringency.	20–30
	Acceptable mouthfeel, slightly sour or sweet, weak *P. lobata* flavor, slight astringency.	10–19
	Poor mouthfeel, unbalanced taste, almost no *P. lobata* flavor, strong astringency.	0–9

## Data Availability

The data presented in this study are available on request from the corresponding author.

## Supplementary Materials

The following supporting information can be downloaded at: https://www.mdpi.com/article/10.3390/molecules31111798/s1, Figure S1: Arrhenius plots of (a) sensory score and (b) Zeta potential.; Text S1: Preliminary orthogonal screening for formulation ratio optimization.

## Abbreviations

The following abbreviations are used in this manuscript:

XG	Xanthan gum
CMC-Na	Sodium carboxymethyl cellulose
SSC	Soluble solid content
