# Supplementary material for: Research on Formulation Optimization and Storage Stability of Pueraria lobata Compound Beverage: Flavor Analysis and Shelf-Life Prediction
Source: Molecules. 2026 May 23;31(11):1798. doi: 10.3390/molecules31111798 (PMC13257803; doi:10.3390/molecules31111798)
Supplement: Supplementary file 1 [file molecules-31-01798-s001.zip › molecules-4281661-supplementary.pdf]

Supplementary Figure S1

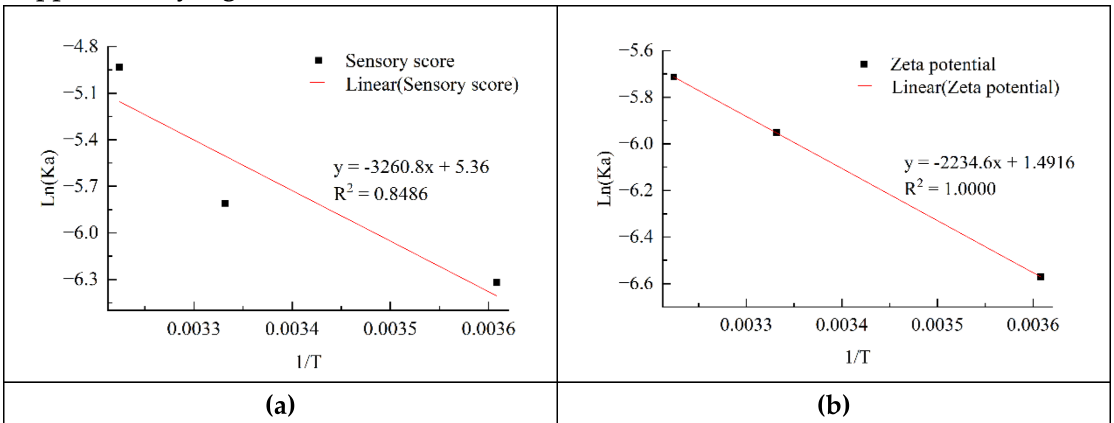

(a)	(b)

Figure S1. Arrhenius plots of (a) sensory score and (b) Zeta potential.

Supplementary Text S1

1 Preliminary orthogonal screening for formulation ratio optimization

To preliminarily screen the formulation ratio of *P. lobata*, *G. lucidum* , *L. barbarum*, and *H. dulcis*, an $L_9(3^4)$ orthogonal design was employed. Four factors (the four plant materials) were investigated, each at three levels. An alcohol-induced HepG2 cell model was used to evaluate the hepatoprotective potential of the candidate formulations, with ALT and AST levels in the culture supernatant used as response indicators. After completion of the nine orthogonal experiments, the formulation showing the strongest inhibitory effect on alcohol-induced ALT and AST elevation was selected as the preferred combination: 1.40 g of *P. lobata*, 12.00 g of *G. lucidum*, 4.00 g of *L. barbarum*, and 13.60 g of *H. dulcis*. This ratio was then used as the starting formulation for subsequent beverage optimization in the present study.
